# Patient Experiences and Treatment Satisfaction with Mini-Dose Glucagon as a Treatment for Hypoglycemia Following Repeated, Prolonged Fasts in Type 1 Diabetes During Ramadan

**DOI:** 10.3390/jcm14093222

**Published:** 2025-05-06

**Authors:** Metab Algeffari, Sufyan Hussain, Mansour Alsharidah, Salsabil Alkhalifah, Turki Almogbel

**Affiliations:** 1 Department of Family and Community Medicine, Collage of Medicine, Qassim University, Buraydah 52571, Saudi Arabia; 2 Department of Diabetes, School of Cardiovascular, Metabolic Medicine and Sciences, King’s College London, London WC2R 2LS, UK; 3 Department of Diabetes and Endocrinology, Guy’s & St Thomas’ NHS Foundation Trust, London SE1 4YB, UK; 4 Institute of Diabetes, Endocrinology and Obesity, King’s Health Partners, London SE1 9RT, UK; 5 Department of Physiology, Collage of Medicine, Qassim University, Buraydah 52571, Saudi Arabia; 6 King Fahd Specialist Hospital, Diabetes and Endocrinology Center, Buraydah 52366, Saudi Arabia

**Keywords:** diabetes mellitus, hypoglycemia, glucagon, fasting, Ramadan, mini dose, complication, risk factors

## Abstract

**Background/Objectives:** Mini—dose glucagon (MDG) is a safe and effective option for managing hypoglycemia during prolonged, repetitive fasts in people with type 1 diabetes (pwT1D) during Ramadan. We aimed in this study to evaluate the effectiveness and patient satisfaction of MDG for the management of fasting—induced hypoglycemia from the perspective of pwT1D fasting during Ramadan. **Methods:** We conducted an online survey shared via social media platforms and email announcements from May 2021 to April 2023 to collect feedback from 136 (72 female) persons with T1D about their experiences using MDG. In the survey, participants were asked to answer questions about diabetes history, hypoglycemia management during Ramadan, the efficacy of MDG treatment, the psychological impact of breaking the fast, side effects, injection experiences, and preferred future treatments for hypoglycemia caused by fasting. **Results:** After using MDG, 123 (91%) of participants reported they were able to complete their fasts. A total of 110 (80%) of participants reported that they prefer to use MDG over oral options in the future to correct fasting—induced hypoglycemia. Furthermore, participants showed significant change in their habits, which would otherwise have left them exposed to hypoglycemia or hyperglycemia for longer periods of time during fasts. **Conclusions:** These data demonstrate positive user experience and satisfaction following MDG as a treatment for fasting—induced hypoglycemia in pwT1D. Furthermore, MDG can promote the successful completion of fasts in Ramadan and encourage a change in unhealthy habits that may lead to prolonged hypoglycemia during fasts.

## 1. Introduction

Despite advancements in treatments and therapies, hypoglycemia remains a significant barrier and a leading cause of morbidity and mortality in individuals with type 1 diabetes (pwT1D) [1]. Various circumstances, such as exercise and fasting, can notably elevate the susceptibility to hypoglycemia in T1D [2,3]. Fasting is an integral part of religious practices across various faiths, including Christianity, Judaism, Islam, Hinduism, and Buddhism. Additionally, intermittent fasting has gained popularity for its potential metabolic health benefits [4]. During Ramadan, over one billion Muslims fast daily from sunrise to sunset, abstaining entirely from food and water [5,6]. Fasting durations in Northern Hemisphere countries can extend from 14 to 20 h, presenting a significant challenge for individuals with T1D. Prolonged fasting during this period can substantially heighten the risk of severe hypoglycemia [7,8]. Nonetheless, studies have shown that a significant proportion of Muslims with type 1 diabetes choose to fast during Ramadan, despite medical recommendations advising against it [8,9].

Given the known potential for glycemic derangements with intensively managed T1D in general, as well as observational data associating significantly higher risks of hypoglycemia with fasting during Ramadan, most international guidelines on diabetes and Ramadan consider T1D as a ‘high’ or ‘very high—risk’ situation where fasting is not recommended [2,10,11]. More recent risk scoring requires further validation, but still leaves a significant proportion of T1D at moderate to high risk [12,13]. However, despite recommendations and alternatives, over 40% of adult Muslims with T1D continue fasting in Ramadan as they feel it essential to their spiritual practice [2], which presents a challenge for healthcare professionals [14].

Observational data demonstrate that the use of technology such as continuous glucose monitoring (CGM) devices with Ramadan—specific education is associated with improvements in the ability to complete fasts, improved glycemic control, and hypoglycemia frequency [15,16]. However, the availability of Ramadan—specific education programs is not widespread, and treatment or avoidance of hypoglycemia primarily relies on breaking the fast. Advances in therapies and technologies have provided avenues to consider safer fasting practices among diabetics; nevertheless, the risks from hypoglycemia still need to be considered and addressed [13].

Mini—dose glucagon (MDG) offers a method to manage hypoglycemia with a subcutaneous treatment. The concept of MDG was first investigated in detail in children showing signs of impending or mild hypoglycemia with age—based doses and a maximum dose of 150 mg [17]. Further data indicated the possibility of using MDG therapy to avoid hypoglycemia during physical activity through the use of a G—Pen Mini with a 150 mg dose of stable glucagon [3]. This treatment was more effective as compared to insulin reduction in avoiding exercise—induced hypoglycemia and avoided significant hyperglycemia noted with 40 mg dose of oral glucose (OG) whilst being as effective in hypoglycemia prevention [3].

According to a widely accepted religious–medical International Consensus, intramuscular and subcutaneous injections do not invalidate Ramadan fasts, while oral or inhaled medications do [18,19]. Therefore, the use of MDG can effectively manage mild or impending hypoglycemia during Ramadan without necessitating breaking the fast and circumventing associated psychological implications. The evidence and experience of MDG use in other contexts provides a strong theoretical basis for the efficacy, safety, and patient satisfaction in this setting. Algeffari et al. (2022) suggest in their study that MDG may be effective in treating hypoglycemia in pwT1D, especially during extended fasting in Ramadan [9]. According to the findings, MDG significantly elevates blood glucose levels by 30 min and 1 h from baseline, compared to oral glucose. Furthermore, despite fasting for at least 8 h or more, MDG continued to deliver effective therapeutic results, as evidenced by significantly elevated blood glucose levels. Moreover, MDG administration over a two—week period was associated with a decrease in the frequency of blood sugar levels below 70 mg/dL and an increase in duration spent within the range of 70–180 mg/dL blood glucose. Additionally, the investigation revealed that participants administered MDG demonstrated a higher success rate in completing fasts when compared to those receiving oral glucose. These findings furnish compelling empirical support for the endorsement of MDG as an effective methodology for the management of hypoglycemia within the type 1 diabetes demographic during the fasting period of Ramadan.

The theoretical basis for the effectiveness, safety, and patient satisfaction in this context is supported by evidence and experience of MDG use in other situations, which received significant engagement on social media with over 92,000 impressions [9,20]. People expressed profound relief at the thought that diabetic patients would no longer have to break their fasts during Ramadan due to this development. Since the study protocol was of great interest to the public, the authors released general information about the study protocol on a pinned tweet for people with diabetes and healthcare professionals [20]. A separate document with information for healthcare providers and a video in Arabic on how to use the injection were available [21]. No use considered against medical advice was announced. In this, it was explained that home use of MDG was a treatment method that needed to be prescribed by your treatment physician. However, current recommendations advocate breaking the fast and treating hypoglycemia with the oral ingestion of glucose or high glycemic—index foods if glucose levels are low as a preferred method.

Within a few weeks, individuals started sharing their personal experiences of using MDG at home to address hypoglycemia induced by fasting. In this study, we collected real—world data on the effectiveness and acceptability of home use of MDG for managing hypoglycemia in pwT1D during Ramadan. Our objective was to evaluate its utility in this unique context and explore its potential applicability to other situations involving prolonged fasting.

## 2. Methodology

**Figure 1:**
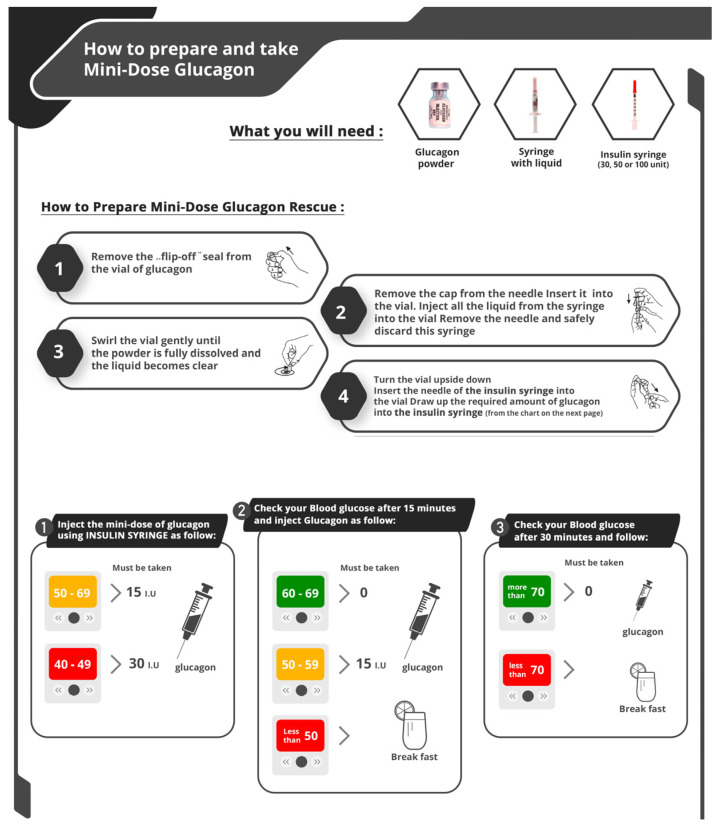
How to prepare and use of MDG as a treatment for fasting—induced hypoglycemia.

Home use of MDG as a treatment for fasting—induced hypoglycemia in pwT1D during Ramadan began to be routinely offered by some healthcare providers following the publication of our protocol in 2020 (Figure 1). Additionally, some pwT1D started adopting this approach independently, without medical advice, as they sought to maintain their fasting during Ramadan.

To evaluate the effectiveness and user experience of MDG, patient responsive objective feedback between May 2021 and April 2023 was collected and evaluated (*n* = 136). Participants were adults aged 18 years or older, with a physician—confirmed diagnosis of type 1 diabetes, and they provided voluntary consent to participate. Individuals who were under 18 years of age, those with other types of diabetes, and those who did not fast during Ramadan were excluded. Feedback was gathered through an online survey, which was shared via social media platforms and email announcements. Prior to data collection, all participants gave informed consent to ensure ethical compliance. Participants were invited to complete a brief 10 min online survey designed to capture their experiences with MDG. The survey process was conducted entirely online, including initial eligibility screening, informed consent, and questionnaire completion. The survey collected comprehensive data on participants’ diabetes history, their experiences managing hypoglycemia during Ramadan, the efficacy of MDG as a treatment of fasting—induced hypoglycemia, the psychological impact of breaking their fast, side effects encountered, their experiences with MDG injections, and their preferences for future treatment methods for fasting—induced hypoglycemia.

As part of the statistical analysis, SPSS 20.0 software (IBM© Corp., Armonk, NY, USA, 2021) was used. Data are presented as mean ± SD for continuous variables, and as numbers and percentages for categorical variables based on the analysis. Qualitative responses, such as “consuming an extra portion at Sahour”, were systematically coded into binary (yes/no) or categorical variables to facilitate quantitative analysis. Open—ended responses were analyzed using thematic analysis, during which the research team identified and categorized key themes emerging from the data. Chi—square tests were conducted to assess associations between categorical variables, with the level of significance set at *p* < 0.05.

## 3. Results

The respondents consisted of 136 pwT1D (72 female). The mean age of subjects was 26.7 ± 9.2 years and had a median diabetes duration of 8.74 ± 6.31 years. A total of 119 (87.5%) of them were using multiple daily injections (MDI) and 17 (12.5%) were using continuous subcutaneous insulin infusion (CSII) therapy. A total of 43 (32%) on CGM devices, while 8.8% had a severe hypoglycemia and 6.6% had diabetic ketoacidosis (DKA) in the last twelve months. Baseline demographic data are shown in (Table 1).

In assessing Ramadan habits among surveyed participants, surveys showed around 69.9% of pwT1D eat extra portions at morning meal (Sahour) to raise their blood sugar above the target to avoid hypoglycemia during fasting hours. Furthermore, 72.8% avoid activity and 64.7% try to keep their blood sugar above the target during the day to avoid hypoglycemia which would otherwise lead them to break their fast. In terms of managing hypoglycemia during fasting hours, over three—quarters (76.5%, 75% consecutively) of pwT1D delay correcting their hypoglycemia if it occurs within 1 h prior to Iftar in order to preserve their fasts. Furthermore, 37.5% pwT1D feel guilt and shame in correcting hypoglycemia in public places (Table 2 and Table S1).

**Figure 2:**
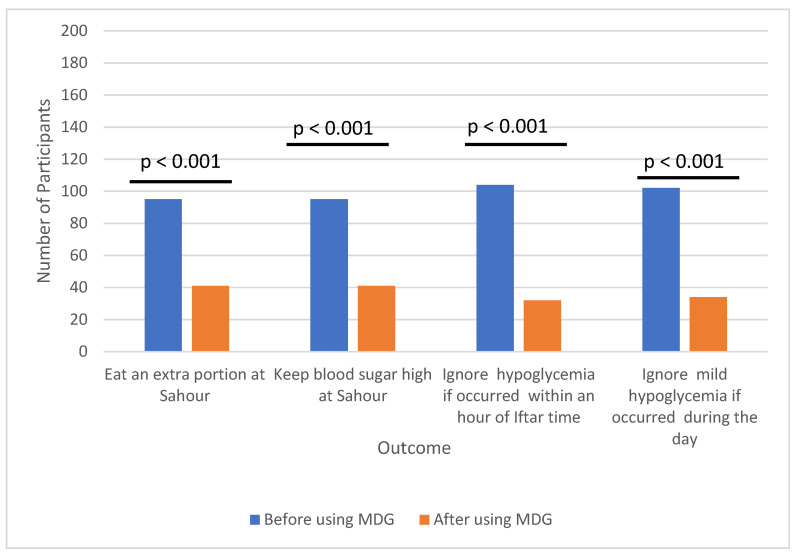
Comparison of study participants habits in managing hypoglycemia during fasting before and after using MDG.

After using MDG, participants reported significant changes in their habits. Over half no longer consumed extra portions of food during the pre—dawn meal (Sahour) to raise their blood sugar above target levels in an effort to prevent hypoglycemia (*p* < 0.001). Additionally, more than half began addressing previously ignored episodes of mild hypoglycemia, even if it occurred within one hour of the evening meal (Iftar) (*p* < 0.001) (Figure 2) (Tables S2 and S3).

A total of 123 participants (91%) successfully completed their fasts (*p* < 0.001), while only 13 (9%) reported needing to break their fast due to improper injection technique or recurrent hypoglycemia. Moreover, 110 participants (80%) expressed a preference for using MDG in the future to manage fasting—induced hypoglycemia (*p* < 0.001). No statistically significant differences in outcomes were observed between subgroups defined by gender, education level, duration of diabetes, therapy type (MDI vs. CSII), or CGM use.

Adverse effects reported by participants included nausea, burning sensations, and injection—site discomfort. Nausea was experienced by 22% of participants, with 11% describing it as intolerable. Burning sensations and injection—site discomfort were reported by 27.2% and 29.4% of participants, respectively, though only 2.2% and 7.4% found these effects intolerable. Importantly, no serious adverse events were reported (Table 2 and Table S4).

A qualitative analysis of participant feedback highlighted several recurring themes. A majority of participants appreciated that MDG allowed them to correct hypoglycemia without needing to break their fast, which was particularly valued during Ramadan. Many also noted that the treatment helped them avoid the feelings of guilt often associated with breaking their fast. Additionally, participants praised the speed and effectiveness of MDG in resolving hypoglycemia and restoring blood sugar levels. However, some expressed dissatisfaction with the complexity of the glucagon preparation technique, signaling an area for potential improvement in future designs.

## 4. Discussion

Fasting during Ramadan holds profound cultural and religious significance for many individuals with type 1 diabetes, presenting both unique challenges and opportunities for patient—centered care [2,22]. We recognize the deep respect owed to these traditions and the autonomy of individuals who choose to fast, even in the face of medical contraindications due to increased risks of hypoglycemia and other complications [10]. In this context, the role of scientific research is twofold: to advance our understanding of the physiological impacts of fasting in T1D and to inform strategies that can help those wishing to observe their faith do so as safely as possible. To provide comprehensive context, it is essential to summarize current international guidelines for managing hypoglycemia in people with T1D during Ramadan. The International Diabetes Federation (IDF) and the Diabetes and Ramadan International Alliance (DAR) have issued evidence—based recommendations that underscore the importance of pre—Ramadan assessment, structured education, and individualized management plans for individuals with diabetes who intend to fast [23,24]. These guidelines generally classify pwT1D as high risk for fasting, primarily due to their increased susceptibility to glycemic variability and hypoglycemia [9]. Nevertheless, for those who opt to fast, structured education delivered by healthcare professionals is paramount. Such programs should encompass meal planning, physical activity, regular blood glucose monitoring, and the timely management of acute complications [8,24]. Additionally, the guidelines provide specific recommendations for insulin dose adjustments and administration timing tailored to observed glucose patterns [8]. This typically involves reducing bolus insulin before Iftar (the evening meal) and/or adjusting basal insulin to minimize hypoglycemia risk, with careful monitoring of post—Iftar glucose to avert hyperglycemia. The use of CGM is strongly advocated, as it enables the real—time tracking of glucose trends, allowing pwT1D to anticipate and address hypoglycemic episodes proactively through timely modifications to insulin therapy and dietary intake [23]. Importantly, it should also be noted that the tendency to overeat after prolonged fasting is a common concern and can pose further health risks, including postprandial hyperglycemia and weight gain [2,23]. This underscores the importance of patient education, individualized care plans, and close medical supervision for pwT1D who wish to observe fasting traditions. By integrating these recommendations, our study addresses current challenges in clinical practice and highlights the potential role of MDG as an alternative strategy for hypoglycemia management in pwT1D during Ramadan.

According to our data, glucagon provides a useful option to treat fasting—induced hypoglycemia in a home setting for adults with type 1 diabetes who are fasting during Ramadan. Previous studies have demonstrated a potential advantage of using glucagon in fasting—induced hypoglycemia, children and during exercise, yet this is the first real—world evaluation that presents data on using MDG for hypoglycemia in prolonged, repetitive fasting situations [3,9,17]. Therefore, this study provides further rationale and validity for using glucagon as a treatment for hypoglycemia, which is likely to be effective in settings following prolonged fasts such as nocturnal hypoglycemia, individuals with T1D undertaking intermittent fasting, or exercising in the fasted state. In the setting of Ramadan fasts, this treatment option helped support people with T1D to complete more fasts successfully and was often preferred over oral treatment. However, it is important to recognize that the self—administration of MDG may carry risks associated with improper injection technique, inadequate aseptic conditions, and potential needle reuse. While MDG offers the advantage of rapidly correcting hypoglycemia without interrupting the fast and may be preferable in certain contexts, it requires appropriate patient education and training to minimize these risks. In contrast, oral treatments are easier to administer and do not involve injection—related concerns, but they necessitate breaking the fast, which may be less acceptable to some individuals. A balanced consideration of these advantages and disadvantages is essential when selecting the most appropriate treatment for hypoglycemia during fasting.

Our data demonstrate that 75% of participants report avoidance of breaking their fasts close to Iftar (evening meal) if they were experiencing hypoglycemia. A total of 75% of participants also avoided correcting any mild hypoglycemia during fasting hours. This may also explain that managing fasting—induced hypoglycemia by ingestion carbs would delay treatment and leave pwT1D exposed to longer duration of hypoglycemia. MDG would reduce these delays by supporting the continuation of fasting.

While nausea without vomiting has been reported infrequently in previous studies after exposure to 150 mg of MDG (occurring in 2 out of 15 participants [3], 3 out of 17 participants [25], and 6 out of 17 participants [9]), the participants in this study reported similar complaints, with 30 out of 136 participants reporting nausea. This effect is likely due to glucagon’s established role in relaxing gastrointestinal smooth muscle [26], although half of the participants described the discomfort as tolerable.

Moreover, the glucagon injection preparation and administration technique used in this study raised concerns, consistent with findings from previous research (16). Despite these challenges, 81% of participants preferred MDG over carbohydrate ingestion as a treatment option during fasting in Ramadan. This preference can be attributed to the perceived convenience of MDG, the lower levels of discomfort compared to breaking the fast to consume carbohydrates, and the psychological benefit of successfully completing the fast.

To address concerns about the injection technique, the development of a reusable device, such as a pen injector, could enhance ease of use and reduce inconsistencies and discomfort. A comparable example is the ready—to—use G—Pen Mini glucagon (Xeris), which remains stable for several years and could serve as a model for improving MDG delivery systems in the future.

It is important to note that physical activity levels are generally reduced during Ramadan, which may influence the frequency and severity of hypoglycemic episodes observed in this study. As such, caution should be exercised when extrapolating these findings to individuals with type 1 diabetes outside of the Ramadan context or in settings where physical activity is not similarly restricted. Previous studies have shown that daily routines, including physical activity and dietary patterns, change significantly during Ramadan, often resulting in decreased energy expenditure and altered glycemic control [7,27]. Individuals who are not participating in Ramadan, or who engage in higher levels of physical activity, may require different management strategies and should seek appropriate medical supervision when considering the use of MDG or other treatments for hypoglycemia. This limitation underscores the importance of individualized care and highlights the need for further research in more diverse settings.

Participants in this survey were representative of a typical clinical cohort from the Middle East clinics and clinics across the globe, which reflect real—world settings in which physicians practice. This group would generally be classified as moderate to high risk for fasting, rather than very high risk [2,10,11]. However, several limitations of the study design must be acknowledged. Firstly, the data relied on self—reported questionnaires, which introduces the potential for bias and inaccuracies. Biochemical and glycemic data such as glycated hemoglobin, fasting blood glucose, and glucagon levels could not be collected or analyzed as part of the study, and satisfaction with hypoglycemia treatment was primarily based on self—reported, unverified glucose changes from baseline to 30 min, measured either by self—monitored blood glucose devices or CGM systems. Furthermore, there was a significant degree of heterogeneity in the intervention, particularly regarding participants’ levels of education about hypoglycemia management and their familiarity with glucagon injection techniques.

In addition, the use of social media for survey distribution may have introduced selection bias and limited the generalizability of our findings. The absence of in—person clarification increases the possibility of misinterpretation of survey questions, potentially affecting the accuracy of responses. The reliance on self—reported data further introduces the risk of recall bias and limits the ability to independently verify clinical events, such as episodes of diabetic ketoacidosis (DKA). Specifically, self—reported DKA events were not validated through medical records, and some participants may not have fully appreciated the seriousness of these episodes.

Despite these limitations, the findings align with prior observations and provide strong support for the utility of this treatment modality in managing fasting—induced hypoglycemia during Ramadan. These results may also be applicable to other forms of religious and non—religious fasting. These results may also have broader applicability to other forms of religious and non—religious fasting. Future research should incorporate objective data collection methods and validated survey instruments to enhance the reliability and validity of findings.

Future work should prioritize developing educational recommendations for individuals using glucagon—based strategies to optimize the replacement of carbohydrate stores following glucagon use. Collaboration with industry to improve drug—delivery devices, such as simplifying injection techniques, is also crucial. While education on glucagon use was not a significant concern in this study, those adopting low—carbohydrate diets alongside intermittent or Ramadan fasting may require additional guidance. Moreover, further research is needed to assess the efficacy of this approach in individuals with low body mass index, hypoglycemia unawareness, or liver and renal impairments.

To conclude, we summaries that MDG administration is an acceptable treatment option for treating hypoglycemia following prolonged fasts in home settings. With prior data establishing its efficacy and safety [9], this study adds further support to its use in this setting. As new methods of delivering and administering glucagon are created, including bihormonal hybrid closed—loop devices, these data offer justification and rationale for using glucagon in managing hypoglycemia during fasting periods in individuals with T1D. Its use supports the successful completion of prolonged fasts in people with T1D during Ramadan. The study demonstrated acceptable tolerability and participants’ preference given that glucagon treatment may avoid the cessation of fasting and therefore also promote safer fasting. Further work is needed to test in very high—risk groups and improve the comfort and usability of devices.

## Figures and Tables

**Table 1:** Participant characteristics.

Parameter	Value (Mean ± SD)/Number (%)
Age (years)	26.7 ± 9.2
Gender (Male/Female)	64 (47%)/72 (53%)
Education status (Secondary/Bachelor)	48 (35.3%)/88 (64.7%)
Duration of diabetes (years)	8.74 ± 6.31
Recent DKA in the last 12 months	9 (6.6%)
Severe hypoglycemia in the last 12 months	12 (8.8%)
Participants using MDI therapy	119 (87.5%)
Participants using CSII therapy	17 (12.5%)
Participants using CGM	43 (32%)

**Table 2:** Comparison of study participants habits before and after using MDG.

Outcome		Before Using MDGN (%)	After Using MDGN (%)
Eat an extra portion at Sahour		95 (69.9)	41 (30.1)
Keep blood sugar high at Sahour		95 (69.9)	41 (30.1)
Avoid activity during the day		99 (72.8)	37 (27.2)
Keep blood sugar high during the day		88 (64.7)	48 (35.3)
Ignore hypoglycemia if occurred within an hour of Iftar time		104 (76.5)	32 (23.5)
Ignore mild hypoglycemia if occurred during the day		102 (75)	34 (25)
Afraid of hypoglycemia during the day in the workplace		55 (40.4)	11 (8.1)
I feel shame when breaking the fast to correct hypoglycemia in public places		51 (37.5)	11 (8.1)
Side effects	Yes, but tolerable	Yes, not tolerable	No
Injection—site discomfort	30 (22)	10 (7.4)	96 (70.6)
Nausea	15 (11)	15 (11)	106 (78)
Burning sensation at injection site	34 (25)	3 (2.2)	99 (72.8)

## Data Availability

Data can be obtained from the correspondence authors upon a reasonable request.

## Supplementary Materials

The following supporting information can be downloaded at: https://www.mdpi.com/article/10.3390/jcm14093222/s1, Table S1: Participant behavior during fasting; Table S2: Participant feedback and experiences of MDG use; Table S3: Participant feedback and experiences of MDG use; Table S4: Common side effects.

## Abbreviations

The following abbreviations are used in this manuscript:

pwT1D	people with type 1 diabetes
MDG	Mini—dose glucagon
OG	oral glucose
CSII	continuous subcutaneous insulin infusion
CGM	continuous glucose monitoring
DKA	diabetic ketoacidosis
