# Supplementary material for: Patient Experiences and Treatment Satisfaction with Mini-Dose Glucagon as a Treatment for Hypoglycemia Following Repeated, Prolonged Fasts in Type 1 Diabetes During Ramadan
Source: J Clin Med. 2025 May 6;14(9):3222. doi: 10.3390/jcm14093222 (PMC12072777; doi:10.3390/jcm14093222)
Supplement: Supplementary file 1 [file jcm-14-03222-s001.zip › jcm-3578807-supplementary.pdf]

Supplemental Material

Supplemental Table S1: Participant behavior during fasting:

	Item A	No	Yes, during OG period	Yes, during MDG period
1	To avoid hypoglycemia during fasting hours, I eat larger amounts (of food) than usual during pre-dawn meal (Sahur time).	O	O	O
2	To avoid hypoglycemia during fasting hours, I tried to keep my blood sugar higher than usual during the pre-dawn meal (Sahur time).	O	O	O
3	To avoid hypoglycemia during fasting hours, I tried to keep my blood sugar higher than usual during fasting hours.	O	O	O
4	To avoid hypoglycemia during fasting hours, I tried to avoid any physical activities during fasting hours.	O	O	O
5	I didn’t break the fast if I had hypoglycemia during the last one hour of fasting.	O	O	O
6	During fasting hours, I didn’t break the fast if I had a mild or asymptomatic hypoglycemia.	O	O	O
7	During fasting hours, I fear of hypo in my workplace.	O	O	O
8	During fasting hours, I feel shame from breaking the fast to correct hypoglycemia in public places	O	O	O

Supplemental Table S2: Participant feedback and experiences of MDG use:

Item C	MDG	Eat or drink any kind of carbohydrates
What would you like to use in the future to correct hypoglycemia during fasting?	O	O

Supplemental Table S3: Participant feedback and experiences of MDG use:

Item E	Answer
What do you like in using Mini-dose Glucagon?	
What do you dislike in using Mini-dose Glucagon?	

Supplemental Table S4: Common side effects:

Item B	No	Yes, but can be tolerated	Yes, but can’t be tolerated
Have you felt any nausea while using Mini-dose Glucagon	O	O	O
Have you felt any discomfort in the injection site while using Mini-dose Glucagon	O	O	O
Burning sensation at injection site	O	O	O
